# Identification of gefitinib off-targets using a structure-based systems biology approach; their validation with reverse docking and retrospective data mining

**DOI:** 10.1038/srep33949

**Published:** 2016-09-22

**Authors:** Nidhi Verma, Amit Kumar Rai, Vibha Kaushik, Daniela Brünnert, Kirti Raj Chahar, Janmejay Pandey, Pankaj Goyal

**Affiliations:** 1 Department of Biotechnology, School of Life Sciences, Central University of Rajasthan, Bandarsindri, NH-8, Kishangarh, Ajmer, Rajasthan 305 817 India; 2 Comprehensive Cancer Center Mainfranken, University Hospital of Würzburg, Versbacher Str. 5, D-97078, Würzburg, Germany

## Abstract

Gefitinib, an EGFR tyrosine kinase inhibitor, is used as FDA approved drug in breast cancer and non-small cell lung cancer treatment. However, this drug has certain side effects and complications for which the underlying molecular mechanisms are not well understood. By systems biology based *in silico* analysis, we identified off-targets of gefitinib that might explain side effects of this drugs. The crystal structure of EGFR-gefitinib complex was used for binding pocket similarity searches on a druggable proteome database (Sc-PDB) by using IsoMIF Finder. The top 128 hits of putative off-targets were validated by reverse docking approach. The results showed that identified off-targets have efficient binding with gefitinib. The identified human specific off-targets were confirmed and further analyzed for their links with biological process and clinical disease pathways using retrospective studies and literature mining, respectively. Noticeably, many of the identified off-targets in this study were reported in previous high-throughput screenings. Interestingly, the present study reveals that gefitinib may have positive effects in reducing brain and bone metastasis, and may be useful in defining novel gefitinib based treatment regime. We propose that a system wide approach could be useful during new drug development and to minimize side effect of the prospective drug.

Lung cancer is the most common cancer as evident from a comprehensive global report that also showed ∼1.8 million new cases reported in 2012^1^. It has been one of the leading causes of cancer-related mortality worldwide (19.4% of all cancers). Additionally, it is more prominent in developing countries (58%) than in developed countries^1^. The abnormal activation of epidermal growth factor receptor (EGFR) tyrosine kinase is responsible for promoting various tumor types, including lung cancer and breast cancer either via an increase in the levels of extracellular ligand, hetero-dimerization of EGFR or its mutational activation^2^,^3^. The most common EGFR mutations reported so far in the case of non-small cell lung cancer (NSCLC) are the deletion of exon 19 and substitution mutation (L858R) at exon 21, leading to constitutive tyrosine kinase activity independent of ligand binding^4^.

Considering the role of EGFR in tumor progression, targeting it for NSCLC treatment is an effective approach. In this direction, various small molecule tyrosine kinase inhibitors, such as erlotinib, gefitinib, lapatinib have been developed and are being used as US Food and Drug Administration (FDA) approved drugs in breast cancer and NSCLC treatment regime^5^. Gefitinib has emerged as a novel therapeutic molecule impairing the tyrosine kinase activity of EGFR effectively. This impairment leads to blockage of downstream signaling and thus inhibits the tumor proliferation activity of EGFR^6^,^7^,^8^. This drug is administered orally at a dosage of 250–500 mg/day and is implemented as first-, second- and third -line therapy in cases of NSCLC^9^.

Gefitinib has certain side effects such as nausea, vomiting, diarrhea and interstitial lung disease^10^. These adverse side effects may be accounted by inhibition of either EGFR and/or drug off-targets^11^. Therefore, analyzing the off-targets of this drug will prove to be effective to reveal the true scenario of Gefitinib: aids and ills and will help in rational modifications of this drug to minimize the side effects. Additionally, for successful establishment of highly efficient drug-based therapies, early identification of adverse drug effects can be a crucial step as up to 40% of drug failures occur during development, adverse events in pre-clinical trials and pharmacokinetics^12^.

Identification of drug off-targets by an *in vitro* counter screening of compounds against numerous receptors and enzymes is expensive and time-consuming^13^,^14^. In contrast, *in silico* analysis of drug off-targets is safe, time-efficient, economical and provides a deeper understanding of the molecular mechanisms of protein-drug interactions. It has been shown that based on the establishment of the structure-activity relationship of small molecules, an *in silico* off-target identification can be obtained^15^,^16^. Various structure-based tools for comparing binding sites of small ligands of distantly related proteins have been developed^17^.

In the present study, we identified gefitinib off-targets using structure-based systems biology approach. We could confirm the binding of identified off-targets with gefitinib using a reverse docking approach. Additionally, through comparative re-docking analyses of identified off-targets with their respective experimentally characterised ligands (ligands that were present in the crystal structure of the protein) and gefitinib, we observed that a few identified off-targets may bind more efficiently with gefitinib compared to their previously reported and experimentally validated ligands. Furthermore, literature survey and data mining has clearly shown several of the identified off-targets were validated in previously reported *in vitro* studies. Together, these observations clearly suggest that off-targets of gefitinib identified in this study might be true off targets and could be involved in the molecular mechanism underlying the possible side effects of this drug. Interestingly, our study suggested not only negative side effects but also positive roles of gefitinib. Additionally, we could identify non-human off-targets that may be used for effective treatment of pathogen-based diseases.

## Results

### Prediction of gefitinib off-targets through Molecular interaction field (MIF) similarity search

**Figure 1:**
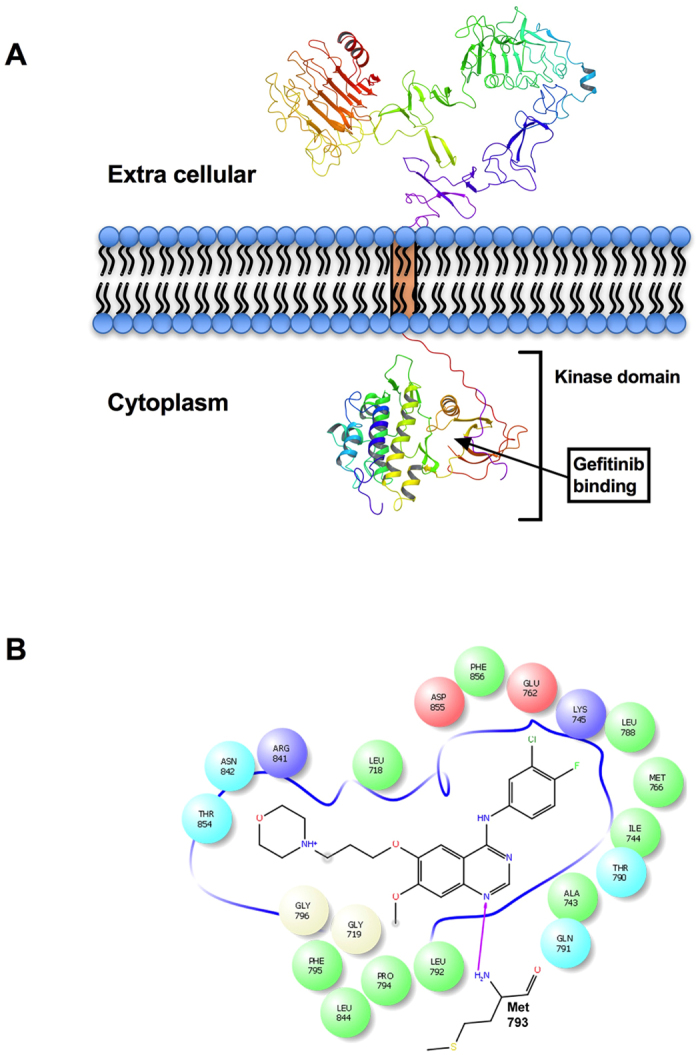
Identification of the Gefitinib binding pocket in EGFR kinase domain. (**A**) Schematic illustration of EGFR receptor on the cell membrane. **(B**) The gefitinib binding pocket was calculated at the distance of 3 Å using Maestro. The identified ligand binding pocket is shown in 2D. Residues are colored according to their properties (red, negatively charged; blue, positively charged; cyan, polar; green, hydrophobic; and white, neutral). The H-bond is shown with magenta arrow.

To carry out the molecular interaction fields (MIFs) similarity search, the crystal structure of EGFR kinase with gefitinib (PDB id: 4WKQ) was used as query structure. This structure showed the binding of different molecules; viz., gefitinib (IRE), 2-(N-morpholino)-Ethanesulfonic acid and sodium ion in the native state. The binding pocket of EGFR kinase around the gefitinib was calculated at the distance of 3 Å and it was found to be defined by residues Leu718, Gly719, Ala743, Ile744, Lys745, Glu762, Met766, Leu788, Thr790, Gln791, Leu792, Met793, Pro794, Phe795, Gly796, Arg841, Asn842, Leu844, Thr854, Asp855 and Phe856 (Fig. 1A,B). Gefitinib interacts with Met793 in the EGFR binding pocket via H-bond formation with the nitrogen atom of the quinazoline ring and Van der Waals interactions.

The MIFs of ligand binding cavities of proteins listed in sc-PDB database (containing 8077 protein structures) were calculated using six properties; H-bond donor/acceptor, aromatic, hydrophobic and positively/negatively charged interactions and compared with query MIF (i.e. Gefitinib binding pocket of EGFR). All the analyzed protein structures were ranked and arranged according to the Tanimoto scores (designated as MIF ranking; Supplementary Table S1). In total, 128 protein structures were found to have Tanimoto scores of ≥0.35 value. These were considered as putative off-targets of Gefitinib, and were selected for further analysis. These selected protein structures represent 50 proteins (Table 1). These hits belong to following species; human (41), rat (3), *Xenopus laevis*, *Pseudomonas putida, Toxoplasma gondii, Cryptosporidium parvum, E. coli*, *Betula pendula* and *Zea mays* (1 each). For subsequent analysis, we focused on the 41 identified-human proteins (Table 1). Most of the putative off-target hits (107) belong to protein kinase family (pfam ID: PF00069 and PF07714). The remaining hits belong to pfam domain Ephrin type-A receptor 2 transmembrane domain, EF-hand domain, Cartilage oligomeric matrix protein, transforming growth factor beta type I GS-motif, MAATS-type transcriptional repressor, Dihydroorotate dehydrogenase, Protein Kinase C terminal domain etc. The pfam ids of above domains are listed in Table 1. Furthermore, the known functions and subcellular localization of the selected proteins are shown in Table 1.

As anticipated, the top ranked structure in MIF search analysis was found to be that of mutated EGFR kinase domain (G719S/T790M) in complex with gefitinib. This data indicates that the method used for binding pocket similarity analysis is appropriate and quite accurate. The other top-ranked structures such as Serine/threonine-protein kinase Chk1 (CHEK1; MIF ranked 2) in complex with 3-(Indol-2-yl) indazoles and Mitogen activated protein kinase-14 (MAPK14; MIF ranked 3) bound with an inhibitor 4-[3-methylsulfanylanilino]-6,7-dimethoxyquinazoline (PDB IDs: 2HOG and 1DI9, respectively) show high similarity to EGFR binding site and might the true off-target proteins.

**Figure 2:**
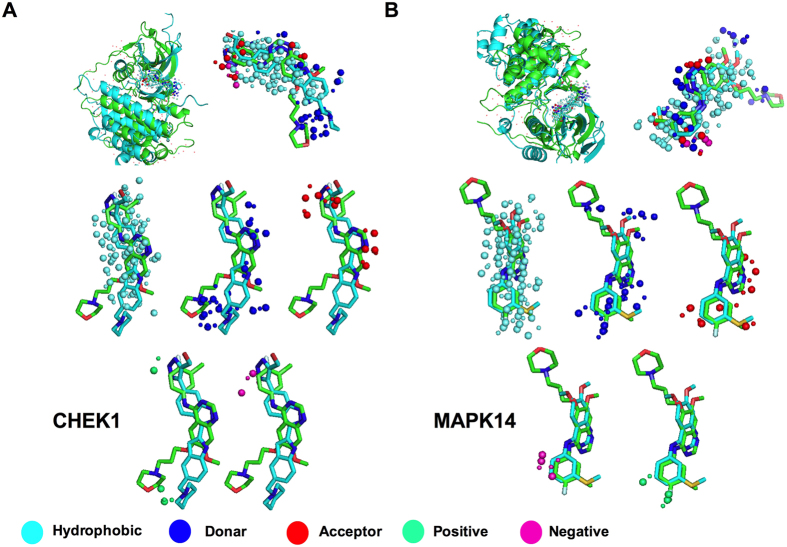
Binding site structure similarity between query protein and off-targets CHEK1 and MAPK14. The superimposed structures of query and off-target proteins in complex with respective ligands are shown (**A,B**; top, left panel). The similarity of binding pockets in query (green) and off-targets (cyan) are shown (**A,B**; top, right panel). The hydrophobic, donor, acceptor, negative and positive binding site probes are shown separately and are represented by cyan, blue, red, magenta and green colored spheres, respectively (**A,B**; middle and lower panel). Large spheres represent the query binding probe while smaller ones represent the off-targets binding probe.

The superimposed structures obtained from the detailed MIF analysis of query (EGFR) and top ranked off-targets (CHEK1 and MAPK14) are shown for five probes (color spheres; Fig. 2). The probes of query and off-target proteins are shown by bigger and smaller spheres, respectively. There are abundant hydrophobic probes surrounding ligands of both query protein and off-targets (cyan spheres in Fig. 2A,B). H-donor probes (blue sphere) of query and off-targets surround the nitrogen (N)-containing C-rings of the ligands. In case of MAPK14, H-donor residues surrounded O-31 and N-containing C-ring same as N-containing C-rings and F present in side group in gefitinib. Positive and negative probes (green and magenta spheres, respectively) surround N-containing C-ring and S-21 containing C-ring in CHEK1 and MAPK14 bound ligands respectively. This indicates that binding pockets of EGFR and the identified off-targets are very similar in nature and therefore it could be argued that gefitinib might be able to bind with these off-targets efficiently and modulate their functions.

### Identification and characterization of binding pockets of off-targets

For binding pocket estimation, we considered only pocket in which the ligand of respective off-target protein was bound. We defined the pocket at the threshold distance of 4 Å based on ligand proximity that is limited to short interactions. The defined pocket with a score ≥0.5 was considered as highly druggable pocket. After estimation of pockets in 128 off-targets, 120 pockets were found to have druggable probability greater than 0.5. Only 8 pockets had values of less than 0.5 (Supplementary Table S2). Since all the identified-pockets were from the crystal structure and have at least one bound ligand, the predicated druggability value of less than 0.5 could not be ignored. The PockDrug server also calculated 66 physicochemical properties (such as hydrophobicity, polarity, aromaticity etc.) of the pockets and the selected parameters are shown in Supplementary Table S2. The binding pocket volumes ranged from 257.99 to 1766.88.

### *In silico* confirmation of off-targets using ligand-protein docking

**Figure 3:**
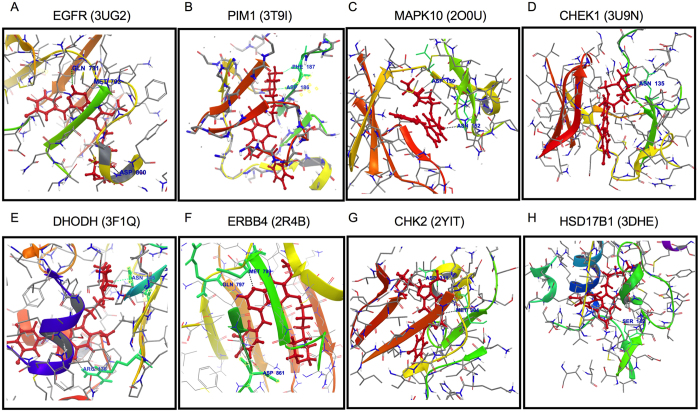
Molecular interactions of gefitinib with selected human off-targets; (**A**) mutant EGFR kinase domain; (**B**) PIM- 1, (**C**) MAPK10; (**D**) CHEK1; (**E**) DHODH; (**F**) ERBB4; (**G**) CHK2 and (**H**) HSD17B1. PDB codes are shown in brackets. The H-bond and polar interactions are represented by black line and green lines respectively.

To further investigate the off-targets identified through MIF similarity searches, we used a reverse docking approach. The docking of gefitinib with each of 128 off-target structures was performed. The docking score was calculated for each gefitinib binding pose that ranges from −1.224 to −12.025, and was subjected to local refinement, binding energy calculations (MM-GBSA method). The 128 structures were re-ranked according to the MM-GBSA binding energy (G-rank; according to lowest binding energies; Supplementary Table S3). Notably, the mutant EGFR kinase domain bound with gefitinib (G179S/T790M; PDB id 3UG2), that was ranked first in MIF similarity search, also showed efficient docking score and binding energy (−6.818 and −77.11 kcal/mol, respectively). The ligand interacts with Met793 and Asp800 via H-bond and Glu791 residues via polar contacts (Fig. 3A). Seven human off-targets (i.e. MAPK10, PIM-1, DHODH, ERBB-4, HSD17B1, CHK2, CHK1) were found to bind with gefitinib with equal or better binding energy than EGFR (Supplementary Table S3). The binding energy for these off-target ranges from −103.446 to −94.712 kcal/mol. The residues involved in binding of gefitinib with these off-targets are shown in Fig. 3B–H.

### Comparison of binding efficiency of identified off- targets with gefitinib and reported ligands

**Figure 4:**
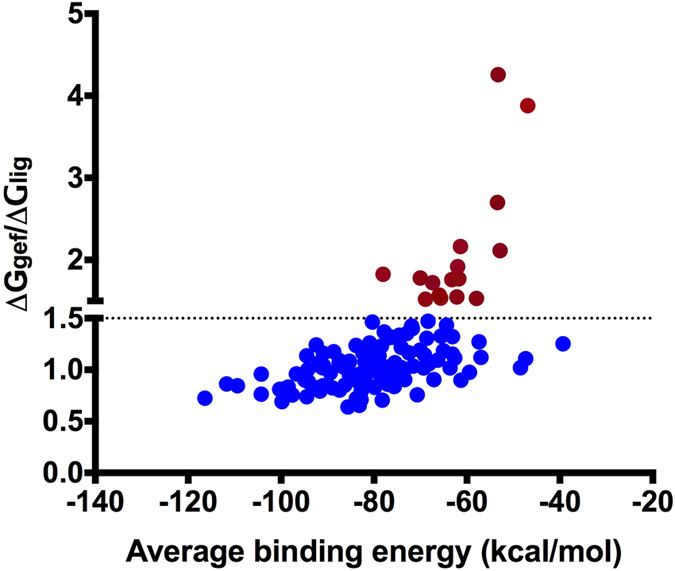
Comparison of the binding energies of gefitinib (∆G_gef_) and the respective bound ligand (∆G_lig_) with the off-target structures. The ratio of gefitinib and reported ligand binding energies (∆G_gef_/∆G_lig_) were plotted against the average binding energy of both. The dotted line shows the threshold ratio 1.5. The spheres with read colour show the significant ratio above the threshold value.

To compare and verify the data, we also performed reversed docking of identified off-targets with their previously reported ligands that were already present in the crystal structure of the off-targets. The docking score and binding energies of these bound ligands are shown in Supplementary Table S3. For determining the difference in binding efficiency of gefitinib ((∆G_gef_) and the respective bound ligand (∆G_lig_) with the off-target structures, the ratio of binding energies (∆G_gef_/∆G_lig_) was calculated and plotted against average binding energies (Fig. 4). The off-targets having ∆G_gef_/∆G_lig_ value ≥ 1.5 were considered to be having significant binding efficiency towards gefitinib compared to their respective reported ligand (Fig. 4, red spheres). In contrast to the bound ligand, gefitinib showed more efficient binding with 15 human and 1 non-human off-target structures (Fig. 4; Table 2). This observation is in agreement with various *in vitro* studies that have reported ligands present in the co-crystals of some of these off-target structures had less efficient binding (IC_50_) compared to other inhibitors used in respective *in vitro* studies (Table 2). This indicates that gefitinib might also be a potent inhibitor of the identified off-targets.

### Retrospective studies of identified off-targets

Various studies have been published on the comprehensive analysis of kinase inhibitors including gefitinib for their selectivity^18^,^19^. The quantitative inhibition data for gefitinib were extracted from previous studies as well as the curated databases DSigDB and ChEMBL^18^,^19^,^20^,^21^. These data were compared with the data obtained from the present study. The results from the above comparison validated the proteins ERBB4, PIM1, MAPK10, MAPK14, ALK, LCK, BTK, ABL1, SRC, STK10, TNK2, KIT, IGF1R, SLK, CHK2, MET, STK17B and SYK as true off-targets of gefitinib (Table 3). These data confirm the specificity of the *in silico* prediction of gefitinib off-targets. Additionally, we also found DHOH, HSD17B1, BMPR1B, NTRK1, ACVRL1, and TTK and proteins as new off-targets of gefitinib that were not included in previous reports (Table 3). Furthermore, we curated the published quantitative inhibition data to identify other off-targets. In total, 22 off-targets that were identified in previous *in vitro* studies however were not found among top 128 hits in our study (Supplementary Table S4). These proteins were also included for further analysis to assess the effects of gefitinib on molecular pathways and diseases.

### Biological pathways analysis

Biological processes were predicted on the basis of gene ontology and the pathways were ranked according to the p-value calculated using Genomatrix software. In total, 971 pathways were found to be significantly correlated with the input off-target genes (Supplementary Table S5). During this analysis, cellular processes such as protein phosphorylation and related pathways were detected in top 50 hits. The cellular proliferation pathways such as cell growth and apoptosis were strongly associated with gefitinib off-targets. Other biological processes, such as cell differentiation, cell communication, stress response, developmental and metabolic process were also found as top hits according to the p-value. The signaling pathways such as MAPK cascade, immune-response-regulating signalling pathway, serine/threonine kinase pathways and neurotrophin TRK receptor signalling pathway were the major pathways that could be correlated with the major reported side effects (Supplementary Fig. S1)^22^.

### Associated disease analysis

**Figure 5:**
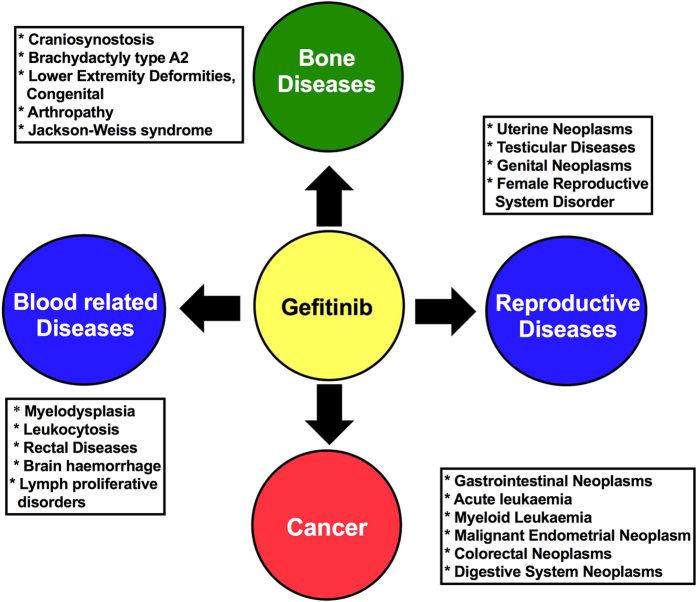
Prediction of clinical diseases that might be modulated by gefitinib-induced side effects.

Clinical diseases prediction is crucial to explain the clinical outcome of the side effects of gefitinib. Sixty clinical diseases were predicted using Genomatrix curated database that were significantly correlated with the gefitinib off-target proteins (Supplementary Table S6). These diseases were group in following broad categories: (i) different cancer types (ii) blood disorders, (iii) bone diseases and (iv) reproductive disorders (Fig. 5). Other discrete diseases/abnormalities of pituitary, endocrine system, hypothalamic, gastrointestinal and bone-marrow were also predicted. These results suggest that gefitinib might have side effects that play a major role in above mentioned diseases.

## Discussion

In the present study, we have carried out a comprehensive analysis of gefitinib off-targets using a systems biology approach; most of the identified off-targets could be validated by retrospective analysis of previously reported studies. In addition, we could also identify a few new off-targets such as DHODH, BMPR1B, NTRK1 and HSD17B1. Together, these observations could be useful for defining the molecular basis of gefitinib-induced side effects and might help in rational improvement of the drug for better treatment.

In our analysis, the mutant EGFR kinase domain in complex with gefitinib interacts with gefitinib through the use of the same residues as the wild type EGFR. Characteristically, the wild type EGFR complexed with an imidazo[2,1-b]thiazole inhibitor (PDBID 3LZB) also showed efficient binding energy with gefitinib. However, the interacting residues were found to be different and had the lowest binding energy. This suggests that the pocket of EGFR kinase domain may adapt to different conformations for interaction with gefitinib.

Interestingly, the top ranked off-targets showed highest binding site similarity but not efficient binding energy. For example, the EGFR to gefitinib docking shows binding energy −88.354 kcal/mol and gefitinib acts as a strong EGFR inhibitor (∼97% inhibition; Ki 0.4 nM (Table 3). Another off-target ERBB4 showed the maximum affinity with gefitinib but was found to be lower ranked in MIF analyses. Notably, ERBB4 is efficiently inhibited by gefitinib (∼76% inhibition; Kd 410 nM) (Table 3). Additionally, other proteins e.g. DHODH and BMPR1B etc. (that were not reported in previous *in vitro* analyses) also showed efficient binding energy with gefitinib. These observations suggest that reverse docking might be a suitable approach for confirming the binding affinities. Previously, it has also been shown that the binding energy calculated on docked poses was useful for predicting the binding affinity of the ligand to the receptor^23^.

During our analyses, a few non-humans off-targets such as src (*Gallus gallus domesticus*), ttgr (*Pseudomonas putida*), aurkb-a (*Xenopus laevis*), ack2 (*Zea mays*), cdpk1 (*Toxoplasma gondii/Cryptosporidium parvum*) and erk2 (*Rattus rattus*) were also found to exhibit efficient binding energy with gefitinib. Amongst the non-human off-targets, ttgr (PDB id: 2UXH), a helix turn helix type transcriptional regulator and antibiotic binding repressor of *Pseudomonas putida* was found to exhibit highly efficient binding with gefitinib (binding energy −100.838 kcal/mol). This observation suggests that gefitinib could be used in potential combination therapy for treatment of antibiotic resistant strains of *Pseudomonas putida*.

Previously reported *in vitro, in vivo* and clinical studies have suggested that gefitinib may induce side effects like pro-apoptosis and cell-cycle inhibition possibly via interacting with off-targets^24^. A recent study has demonstrated that gefitinib is able to induce cardiac hypertrophy through differential expression of apoptotic and oxidative stress genes^25^. In contrast, a few previous studies have also demonstrated positive effects of gefitinib such as bone pain relief during bone metastasis and brain metastasis^26^,^27^,^28^. However, the underline molecular basis of such effects is poorly understood. In the present study, we identified that gefitinib off-targets are associated with different biological pathways that may explain the molecular mechanism of such positive and negative effects of gefitinib. The identified off-targets (ACVR1, DHODH, BTK, FGFR1, EGFR, FGFR2 and CHEK2) are linked with bone diseases. Similarly, an earlier report demonstrating drug resistance against EGFR therapy in rectal diseases and non-small cell lung cancer through dysregulation of EGFR endocytosis can be explained via identified off-targets (LYN, SRC, ABL2, ABL1, SYK, TNK2, MAPKAPK2, GAK and MAPK1) that are involved in endocytosis process.

This study has found the off-targets binding to gefitinib suggesting the molecular mechanisms of the side-effects of this drug. The biological processes, regulated by off-targets are interesting to focus in future studies. Notably, this study also suggested positive roles of gefitinib in the treatment regime. System-wide *in silico* approaches may facilitate the identification of side effects of preclinical and commercial drugs onto the target and off-targets. This strategy may have important applications for rational improvement of drug design and development.

## Methods

### Druggable proteome data set

Sc-PDB database v.2013 (http://cheminfo.u-strasbg.fr/scPDB/) was used as source of druggable proteome for off-target analysis. The data set contains 8077 structures with druggable binding sites that represent 3678 proteins and 5608 different HET ligands^29^.

### Binding site similarity search

To compare physicochemical similarities in binding pockets of different proteins, MIF within binding sites’ volumes and pairwise MIF similarities between binding sites were calculated using the IsoMIF Finder (http://bcb.med.usherbrooke.ca/isomif)^30^. In this study, the crystal structure of EGFR kinase with Gefitinib (PDB id 4WKQ; 1.85 Å) was selected as query protein. The binding pocket at the distance of 3 Å around gefitinib was cropped using GetCleft tool^30^ and subsequently used for MIF similarities search against Sc-PDB database. The parameters used for analysis were as follows: grid spacing 1.5 Å and geometric distance threshold 3.0 Å. Structures were ranked by Tanimoto score and designated as MIF rank. Top 128 hits of gefitinib binding targets were selected for further studies. The details of 128 PDB structures with references were listed in Supplementary Table S8.

### Pocket estimation and characterization

The pockets of top 128 hits were estimated based on ligand proximity within a fixed distance threshold from the bound ligand. To extract the residues localized within threshold distance; “PockDrug-Server” (http://pockdrug.rpbs.univ-paris-diderot.fr) was used. The PDB files were uploaded on the server and “prox” method was selected to estimate the pocket using threshold distance at 4 Å. The ligand information in HET code was also given during prediction^31^.

### Protein-Ligand docking

The potential off-targets-identified from MIF similarity search were further processed for binding analysis of gefitinib and previously characterized respective ligands. The Glide 6.9 ligand-receptor docking program (Schrödinger 10.4; Schrödinger Inc, USA) was used for docking of gefitinib to each off-target structure. The ligand library of gefitinib was prepared by LigPrep tool from Schrödinger program with OPLS-2005 force field. Receptor grid was generated in the vicinity of bound ligand of each identified-off target crystal structure using Glide-Receptor Grid generation tool with default parameters. Ligand docking was performed with extra precision (XP) Glide docking module. The binding energies of docking poses were calculated using MM-GBSA method (Prime, Schrödinger Inc, USA) with default parameters.

### Literature mining and retrospective studies

PubMed and Google Scholar were used to search publications and research studies relevant to gefitinib. These reports were analyzed for determining the selectivity of gefitinib. We also searched DSigDB database, a collection of small molecules including drugs based compounds and their quantitative inhibition data, for the analysis of gefitinib inhibition^32^.

To analyze the biological pathways corresponding to identified off-targets, Genomatrix software (Genomatrix, Munich, Germany) with Gene Ranker and GePS (Pathway system) modules were used. The identified off-targets were used as input query, network pathways were constructed and the identified pathways were ranked according to p-value.

## Additional Information

**How to cite this article**: Verma, N. *et al*. Identification of gefitinib off-targets using a structure-based systems biology approach; their validation with reverse docking and retrospective data mining. *Sci. Rep.* **6**, 33949; doi: 10.1038/srep33949 (2016).

## Supplementary Material

Supplementary Information

## Figures and Tables

**Table 1:** The details of off-targets of gefitinib identified through MIF similarity serach analysis.

S. No.	Gene names	PDB ID	UniprotKB ID	Pfam ID	Organism	Functions	Locations
1	BMPR1B	3MDY	O00238	PF08515	Human	positive regulation of chondrocyte differentiation	cell membrane
2	SRC SRC1	1YOL, 1Y57 3EN5, 3EN7, 3EN4	P12931 P00523	PF07714	Human Chicken	regulation of cytoskeletal organization, activation of EGF-mediated calcium- chloride channel	cell membrane, mitochondrial inner membrane, nucleus, cytoplasm
3	FGFR2 BEK KGFR KSAM	3RI1, 1OEC	P21802	PF07714	Human	regulates the cell proliferation, differentiation, migration, apoptosis, and embryonic development	cell membrane, Golgi apparatus, cytoplasmic vesicles
4	ACVRL1 ACVRLK1 ALK1	3MY0	P37023	PF08515	Human	regulator of normal blood vessel development	cell membrane
5	MAPK8 JNK1 PRKM8 SAPK1 SAPK1C	3ELJ	P45983	PF00069	Human	regulates the circadian clock	cytoplasm, nucleus
6	ALK	2XBA	Q9UM73	ALK_HUMAN	Human	genesis and differentiation of the nervous system	cell membrane
7	Comp	1FBM	P35444	COMP_RAT	Rat	regulates structural integrity of cartilage	extra cellular matrix
8	AURKA AIK AIRK1 ARK1 AURA AYK1 BTAK IAK1 STK15 STK6	3LAU, 3UOH	O14965	PF00069	Human	regulates cell cycle progression	cytoplasm, cytoskeleton, microtubule organizing center, centrosome
9	DMPK DM1PK MDPK	2VD5	Q09013	DMPK_HUMAN	Human	maintains skeletal muscle structure and function	cell membrane, ER membrane, mitochondrial outer membrane
10	SLK KIAA0204 STK2	2J51	Q9H2G2	PF00069	Human	mediates apoptosis and actin stress fiber dissolution	cytoplasm
11	ERBB4 HER4	2R4B	Q15303	PF07714	Human	regulates development of the heart, the central nervous system and the mammary gland, promote apoptosis	cell membrane, nucleus and mitochondria
12	ttgR T1E_0244	2UXH	Q9AIU0	TTGR_PSEPT	*Pseudomonas putida* (strain DOT-T1E)	responsible for antibiotics resistance	not known
13	TNK2 ACK1	3EQR	Q07912	ACK1_HUMAN	Human	involved in trafficking and clathrin-mediated endocytosis	cell membrane, nucleus, endosome
14	EGFR ERBB ERBB1 HER1	3UG2, 3LZB	P00533	EGFR_HUMAN	Human	activates several signal cascades	cell membrane, ER membrane nucleus membrane, Golgi membrane, endosome
15	STK25 SOK1 YSK1	2XIK	O00506	STK25_HUMAN	Human	response against environmental stress	cytoplasm, Golgi apparatus
16	LCK	3KMM, 3KXZ, 3ACK, 3MPM, 3AC3, 3AC2, 3AD4	P06239	LCK_HUMAN	Human	responsible for selection and maturation of developing T-cells	cytoplasm, cell membrane
17	IGF1R	2ZM3	P08069	IGF1R_HUMAN	Human	involved in cell growth and survival control	cell membrane
18	CHEK1 CHK1	2HOG, 2E9N, 4FTI, 4FSR, 2XF0, 2HXL, 4FTQ, 2HY0, 4FT3, 4FTN, 2BRB, 2BRH, 2XEY, 4FTO, 2QHM, 2E9U, 4FT0, 3UN9, 4FTC, 2BR1	O14757	CHK1_HUMAN	Human	required for cell cycle arrest and activation of DNA repair	nucleus, cytoplasm
19	PRKCI DXS1179E	1ZRZ	P41743	KPCI_HUMAN	Human	Plays a protective role against apoptotic stimuli	cytoplasm, membrane, nucleus, endosome
20	MAP3K9 MLK1 PRKE1	3DTC	P80192	M3K9_HUMAN	Human	role in the cascades of cellular responses	intracellular
21	FGFR1 BFGFR CEK FGFBR FLG FLT2 HBGFR	3TT0, 4F64	P11362	FGFR1_HUMAN	Human	essential for regulation of embryonic development, cell proliferation, differentiation and migration	cytoplasm, cell membrane, nucleus, cytoplasmic vesicles
22	MAPK14 CSBP CSBP1 CSBP2 CSPB1 MXI2 SAPK2A	1DI9, 3FLW, 3KF7, 3ROC, 3IW7, 3FLZ, 3MVM, 2ZB1, 2RG6, 1WBW	Q16539	MK14_HUMAN	Human	essential component of the MAP kinase signal transduction pathways responsible for activating the cascades of cellular responses	cytoplasm. nucleus
23	KIT SCFR	3G0E	P10721	KIT_HUMAN	Human	responsible for regulation of hematopoiesis, stem cell maintenance, gametogenesis, mast cell development, and in melanogenesis	cytoplasm, cell membrane
24	STK17B DRAK2	3LM0	O94768	ST17B_HUMAN	Human	positive regulator of apoptosis	cell membrane, nucleus
25	aurkb-a airk2-a	2VRX	Q6DE08	AUKBA_XENLA	*Xenopus laevis*	key regulator of mitosis	nucleus, chromosomes
26	ACK2	1ZOH	P28523	CSK2A_MAIZE	*Zea mays* (Maize)	utilizes acidic proteins	not known
27	ABL1 ABL JTK7	3UE4, 3KF4 (Mouse)	P00519	ABL1_HUMAN	Human	responsible for cell growth and survival	cytoplasm, nucleus, mitochondria
28	MAPK10 JNK3 JNK3A PRKM10 SAPK1B	2O2U, 4Z9L, 3CGO, 2O0U, 3TTJ, 2ZDT	P53779	MK10_HUMAN	Human	involved in neuronal proliferation, differentiation, migration and programmed cell death	cytoplasm. nucleus, mitochondria, membrane
29	MAPKAPK2	3KA0	P49137	MAPK2_HUMAN	Human	involved in cytokine production, endocytosis, reorganization of the cytoskeleton, chromatin remodeling and DNA damage response	cytoplasm, nucleus
30	BTK AGMX1 ATK BPK	3PJ1, 3PJ2, 3OCT	Q06187	BTK_HUMAN	Human	Vital for B lymphocyte development, differentiation and signaling	cytoplasm. nucleus, cell membrane
31	ACVR1 ACVRLK2	3MTF, 3OOM	Q04771	ACVR1_HUMAN	Human	involved for left-right pattern formation during embryogenesis	membrane
32	SYK	1XBC	P43405	KSYK_HUMAN	Human	regulates innate and adaptive immunity, cell adhesion, osteoclast maturation, platelet activation and vascular development	cytoplasm, cell membrane
33	EPHB4 HTK MYK1 TYRO11	2VX1, 2VWU, 2×9F, 2VWX, 2VWW	P54760	EPHB4_HUMAN	Human	important in tumor angiogenesis	cell membrane
34	CDPK1	3T3U, 3NYV, 3MWU, 3MA6	Q9BJF5A3FQ16	Q9BJF5_TOXGO A3FQ16_CRYPI	*Toxoplasma gondii Cryptosporidium parvum* (strain Iowa II)	binds with metals and nucleotides	not known
35	CDK6 CDKN6	3NUX	Q00534	CDK6_HUMAN	Human	controls cell cycle and differentiation	cytoplasm. nucleus
36	DHODH	3F1Q	Q02127	PYRD_HUMAN	Human	catalyzes the conversion of dihydroorotate to orotate	mitochondrial inner membrane
37	CDK2 CDKN2	3EZV, 2UZD, 2IW6, 3R6X, 1DI8, 2VTQ, 2VU3, 2W17, 3R1Q, 1GIJ	P24941	CDK2_HUMAN	Human	control of the cell cycle; essential for meiosis, but non-essential for mitosis	cytoplasm. nucleus, endosome
38	PIM1	3MA3, 2O65, 3R04, 3T9I, 3VBY, 3UMX, 4ENY, 3CY3, 3JY0	P11309	PIM1_HUMAN	Human	involved in cell survival and cell proliferation, provide advantage in tumorigenesis	cytoplasm. nucleus, cell membrane
39	STK10 LOK	4BC6	O94804	STK10_HUMAN	Human	regulates lymphocyte migration	cell membrane
40	Mapk1 Erk2 Mapk Prkm1	2Z7L	P63086	MK01_RAT	Rat	plays important role in the MAPK/ERK cascade	cytoplasm, nucleus
41	PRKACA PKACA	3AMB, 1SZM	P17612 P00517	KAPCA_HUMAN KAPCA_BOVIN	Human Bovine	involved in the regulation of platelets	cytoplasm. nucleus, cell membrane, mitochondria
42	ITK EMT LYK	3T9T, 3V8T	Q08881	ITK_HUMAN	Human	involved in regulation of the adaptive immune response	cytoplasm
43	MET	3ZZE	P08581	MET_HUMAN	Human	mediates entry of the pathogen into cells	membrane
44	CHK2	2YCQ, 2YIT	O96017	CHK2_HUMAN	Human	required for checkpoint-mediated cell cycle arrest	nucleus
45	TTK	3HMO	P33981	TTK_HUMAN	Human	associated with cell proliferation	membrane, spindle
46	NTRK1	4AOJ	P04629	NTRK1_HUMAN	Human	involved in the development and the maturation of the central and peripheral nervous systems	cell membrane, early endosome membrane, late endosome membrane
47	TTR	1KGJ	P02767	TTHY_RAT	Rat	transports thyroxine from the bloodstream to the brain	secreted
48	HSD17B1	3DHE	P14061	DHB1_HUMAN	Human	has 20-alpha-HSD activity	cytoplasm
49	METF	3FSU	P0AEZ1	METF_ECOLI	*E.coli*	converts homocysteine to methionine	cytosol
50	BETVIA	4A84	P15494	BEV1A_BETPN	*Betula pendula*	steroid carrier protein	cytoplasm

**Table 2:** The list of characterized off-targets having significant binding efficiency towards gefitinib compared to their respective reported ligands.

S. No.	Name	PDB	Ligand present in co**-**crystal	gefitinib MM-GBSA binding energy	known ligands MM-GBSA binding energy	∆G_gef_/∆G_lig_	IC50 (nM) of known ligand
1	Cyclin-dependent kinase 2	2UZD	C85	−86.307	−20.274	4.257028707	ND
2	Cyclin-dependent kinase 2	2VTQ	LZA	−74.582	−19.222	3.880033295	ND
3	Fibroblast growth factor receptor 2	3RI1	3RH	−77.977	−28.889	2.699193465	180
4	Ephrin type-B receptor 4	2×9F	UNN	−83.961	−38.83	2.16227144	140
5	STE20-like serine/threonine-protein kinase	2J51	DKI	−71.754	−33.967	2.112462096	140
6	Mitogen-activated protein kinase 14	1WBW	LI4	−81.464	−42.498	1.916890207	2
7	HTH-type transcriptional regulator TtgR	2UXH	QUE	−100.838	−55.277	1.824230693	ND
8	Serine/threonine-protein kinase pim-1	3MA3	01I	−89.67	−50.377	1.779978959	61
9	Bone morphogenetic protein receptor type-1B	3MDY	LDN	−78.885	−44.586	1.769277352	5000 (Kd)
10	Activin receptor type-1	3MTF	A3F	−80.671	−45.83	1.760222562	614 (Kd)
11	Mitogen-activated protein kinase 10	2O2U	738	−85.273	−49.501	1.722652068	400 (Ki)
12	Cyclin-dependent kinase 2	2IW6	QQ2	−80.586	−51.35	1.569347614	44000
13	Insulin-like growth factor 1 receptor	2ZM3	575	−75.531	−48.801	1.547734678	22900
14	Cyclin-dependent kinase 2	2W17	I19	−79.496	−51.79	1.534968141	60
15	Serine/threonine-protein kinase Chk1	2BRH	DFW	−70.09	−45.808	1.530082082	9.7
16	Tyrosine-protein kinase ITK/TSK	3V8T	477	−83.127	−54.685	1.520106062	0.3

**Table 3:** Binding energies of identified off-targets with gefitinib and their comparison with data previously reported in high throughput *in vitro* studies.

S. No.	Name	Gene Name	mmGBSA DG binding energy (kcal/mol)	Anastassiadis et al. (% activity)	Davis et al. (Kd)	Apsel et al. (IC50)	ChEMBL (Ki)
1	Mitogen-activated protein kinase10	MAPK10, JNK3	−103.446	96.64	3200	—	794.33
2	Serine/Threonine-protein kinase pim-1	PIM1	−102.399	86.45	—	—	1995.26
3	Dihydroorotate dehydrogenase (quinone) mitochondrial	DHODH	−100.487	—	—	—	—
4	Receptor tyrosine-protein kinase erbB-4	ERBB4, HER4	−100.195	24.15	410	—	158.49
5	Estradiol 17-beta-dehydrogenase 1	HSD17B1	−97.635	—	—	—	—
6	Serine/Threonine-protein kinase Chk2	CHEK2, CHK2	−94.826	83.95	800	—	630.96
7	Serine/Threonine-protein kinase Chk1	CHEK1, CHK1	−94.712	95.27	—	—	6309.57
8	Tyrosine-protein kinase Lck	LCK	−93.312	68.44	630	—	398.11
9	Hepatocyte growth factor receptor	MET	92.659	—	3500	—	—
10	Mitogen activated protein kinase 14	MAPK14	−91.953	74.59	ND	—	501.19
11	tyrosine-protein kinase BTK	BTK	−90.291	60.56	—	—	1258.93
12	Tyrosine-protein kinase ABL1	ABL1, JTK7	−89.891	86.35	230	1200	630
13	Mitogen-activated protein kinase kinase kinase 9	MAP3K9, MLK1	−89.843	109.52	—	—	—
14	Activin receptor type-1	ACVR1, ALK2	−88.685	105.63	—	—	3981.07
15	Epidermal growth factor receptor	EGFR, ERBB, HER1	−88.354	2.97	1	—	0.4
16	High affinity nerve growth factor receptor	NTRK1	−87.784	—	—	—	—
17	Serine/Threonine-protein kinase 25	STK25	−86.644	97.38	—	—	—
18	proto-oncogene tyrosine protein kinase Src	SRC	−86.531	79	3800	1100	1995.26
19	Cyclin-dependent kinase 2	CDK2, CDKN2	−86.307	100.65	—	—	6309.57
20	Serine/Threonine-protein kinase 17B	STK17B, DRAK2	−84.931	—	3800	—	—
21	protein kinase C iota type	PRKCI	−84.923	—	—	—	5011.87
22	Ephrin type-B receptor 4	EPHB4, HTK, MYK1	−83.961	70.89	2500	1000	
23	Tyrosine-protein kinaseITK/TSK	ITK, EMT, LYK	−83.127	99.94	—	—	—
24	Myotonin protein kinase	DMPK, MDPK	−81.558	90.09	6900	—	—
25	Serine/Threonine-protein kinase10	STK10, LOK	−80.265	38.59	470	—	—
26	Aurora kinase A	AURKA, STK15, STK6	−74.879	95.27	—	—	3162.28
27	Bone morphogenetic protein receptor type-1B	BMPR1B	−78.885	—	—	—	—
28	Serine/Threonine-protein kinase receptor R3	ACVRL1, ALK1	−78.538	96.86	—	—	—
29	ALK tyrosine kinase receptor	ALK	−78.228	98.75	—	—	1258.93
30	Fibroblast growth factor receptor 2	FGFR2, BEK	−77.977	98.73	—	—	—
31	mitogen activated protein kinase 8	MAPK8, JNK1	−77.826	93.07	—	—	—
32	FIbroblast growth factor receptor 1	FGFR1	−76.2	96.4	−	—	3981.07
33	MAP kinase-activated protein kinase 2	MAPKAPK2	−75.845	97.5	—	—	
34	Insulin-like growth factor 1 receptor	IGF1R	−75.531	92.33	—	—	1258.93
35	Activated CDC42 kinase1	TNK2, ACK1	−74.789	72.8	—	—	
36	Mast/stem cell growth factor receptor Kit	KIT, SCFR	−73.003	96.4	1800	—	—
37	STE20-like serine/threonine-protein kinase	SLK, STK2	−71.754	83.71	920	—	398.11
38	cAMP-dependent protein kinase catalytic subunit alpha	PRKACA, PKACA	−69.932	—	—	—	5011.87
39	Mitogen-activated protein kinase 1 (Rat; identical to Human)	MAPK1, ERK2	−69.262	103.1	—	—	
40	Tyrosine-protein kinase SYK	SYK	−65.782	104.18	—	—	1584.89
41	Dual specificity protein kinase TTK	TTK	−64.705				
42	Cyclin-dependent kinase 6	CDK6, CDKN6	−63.744	98.69	—	—	—
