# Supplementary material for: Identification of gefitinib off-targets using a structure-based systems biology approach; their validation with reverse docking and retrospective data mining
Source: Sci Rep. 2016 Sep 22;6:33949. doi: 10.1038/srep33949 (PMC5032012; doi:10.1038/srep33949)

Identification of gefitinib off-targets using a structure-based systems biology approach; their validation with reverse docking and retrospective data mining

Nidhi Verma[1], Amit Kumar Rai[1], Vibha Kaushik[1], Daniela Brünnert[2], Kirti Raj Chahar[1], Janmejay Pandey[1$] and Pankaj Goyal[1*$]

[1]Department of Biotechnology, School of Life Sciences, Central University of Rajasthan, Bandarsindri, NH-8, Kishangarh, Ajmer, Rajasthan 305 817 India

[2]Comprehensive Cancer Center Mainfranken, University Hospital of Würzburg, Versbacher Str. 5, D-97078, Würzburg, Germany

Figure S1: List of the gefitinib side effects reported in the previous studies.

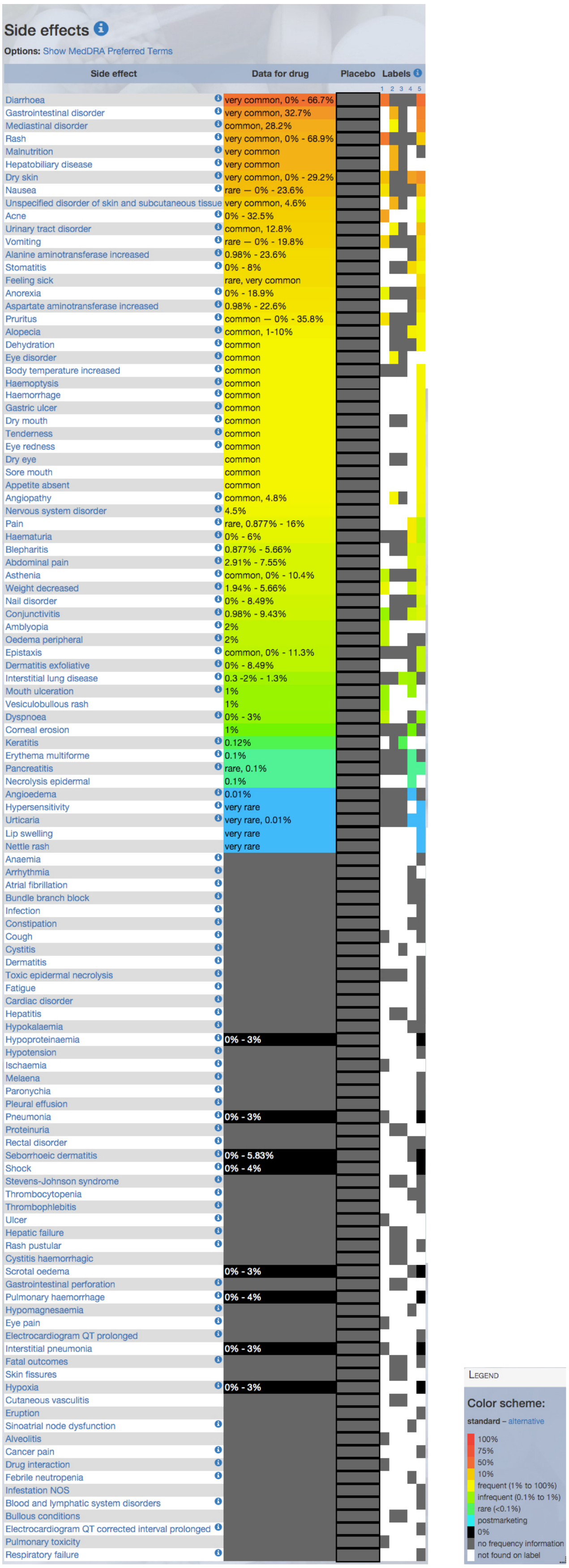

Supplement: Supplementary Information [file srep33949-s1.pdf]
